# Cellulase Linkers Are Optimized Based on Domain Type and Function: Insights from Sequence Analysis, Biophysical Measurements, and Molecular Simulation

**DOI:** 10.1371/journal.pone.0048615

**Published:** 2012-11-06

**Authors:** Deanne W. Sammond, Christina M. Payne, Roman Brunecky, Michael E. Himmel, Michael F. Crowley, Gregg T. Beckham

**Affiliations:** 1 Biosciences Center, National Renewable Energy Laboratory, Golden, Colorado, United States of America; 2 National Bioenergy Center, National Renewable Energy Laboratory, Golden, Colorado, United States of America; 3 Department of Chemical Engineering, Colorado School of Mines, Golden, Colorado, United States of America; University of Waikato, New Zealand

## Abstract

Cellulase enzymes deconstruct cellulose to glucose, and are often comprised of glycosylated linkers connecting glycoside hydrolases (GHs) to carbohydrate-binding modules (CBMs). Although linker modifications can alter cellulase activity, the functional role of linkers beyond domain connectivity remains unknown. Here we investigate cellulase linkers connecting GH Family 6 or 7 catalytic domains to Family 1 or 2 CBMs, from both bacterial and eukaryotic cellulases to identify conserved characteristics potentially related to function. Sequence analysis suggests that the linker lengths between structured domains are optimized based on the GH domain and CBM type, such that linker length may be important for activity. Longer linkers are observed in eukaryotic GH Family 6 cellulases compared to GH Family 7 cellulases. Bacterial GH Family 6 cellulases are found with structured domains in either N to C terminal order, and similar linker lengths suggest there is no effect of domain order on length. *O*-glycosylation is uniformly distributed across linkers, suggesting that glycans are required along entire linker lengths for proteolysis protection and, as suggested by simulation, for extension. Sequence comparisons show that proline content for bacterial linkers is more than double that observed in eukaryotic linkers, but with fewer putative *O*-glycan sites, suggesting alternative methods for extension. Conversely, near linker termini where linkers connect to structured domains, *O*-glycosylation sites are observed less frequently, whereas glycines are more prevalent, suggesting the need for flexibility to achieve proper domain orientations. Putative *N*-glycosylation sites are quite rare in cellulase linkers, while an N-P motif, which strongly disfavors the attachment of *N*-glycans, is commonly observed. These results suggest that linkers exhibit features that are likely tailored for optimal function, despite possessing low sequence identity. This study suggests that cellulase linkers may exhibit function in enzyme action, and highlights the need for additional studies to elucidate cellulase linker functions.

## Introduction

Many proteins are composed of multiple structured domains connected by linker regions [1]. Numerous studies have shown that linkers are often not only flexible connectors, but can facilitate optimal interaction between structured domains [2]–[16]. To explain these types of observations, Nussinov *et al*. hypothesized that linkers are optimized to preferentially sample functionally relevant conformations [17]. They posit that if linkers are overly flexible, there will be a decrease in the functional efficiency of a given protein or enzyme complex due to greater sampling of non-productive domain orientations. While there is evidence of conserved function in linkers, sequence analyses have demonstrated that these regions are highly divergent, and biophysical analyses of select linker peptides have shown that these regions do not exhibit considerable structural elements [18]–[22]. The challenge, therefore, is to identify important features in linker regions and understand the role these features play in protein function.

Linker regions are a subset of intrinsically disordered proteins (IDPs). While linkers vary in composition, they generally show a bias in amino acid content, exhibiting a lack of hydrophobic amino acids that are prevalent in structured protein cores. The bias in amino acid content manifests in sequences with low complexity [23]. Bioinformatics studies have identified putative linker regions based on the presence of IDP hallmarks, such as amino acid composition and sequence complexity [22], [24]. Also, IDPs, including linkers, generally exhibit low sequence conservation, highlighting the lack of conserved interactions that might impart a defined globular structure [25]. Thus, traditional sequence alignments of IDPs are largely ineffective for the assignment of structure-function relationships in contrast to ordered domains wherein putative function can often be assigned based on sequence identity.

In addition to sequence variability, significant variation is also observed in linker lengths, especially when comparing sequences from different organisms. Several studies have demonstrated that the average length of eukaryotic proteins is significantly longer than the average length of prokaryotic proteins, even when considering proteins with similar function [26]–[28]. This observed difference in median protein length between eukaryotes and prokaryotes was ascribed to the linker regions connecting globular protein domains [24]. The variability in average linker lengths between eukaryotes and prokaryotes suggests evolutionary pressure governing conservation in linkers is based on something other than, or in addition to, protein function. The conservation of linker length for cellulases from bacteria and eukaryotes is examined in this study to answer questions such as whether linker length is conserved within glycoside hydrolase (GH) or carbohydrate-binding module (CBM) families, and thus whether linker length is optimized based on enzyme function.

Studies focused on the systematic manipulation of linkers provide insight into identifying conserved features in these regions of low sequence conservation. For example, Robinson *et al*. evaluated the flexible linker connecting two interacting domains from the Arc repressor protein, which is a DNA-binding protein [7]. They showed the stability and folding rate of Arc could be optimized by varying the length and amino acid content of the linker region. Importantly, they randomized the linker sequence with little effect on the physical properties of the protein, demonstrating that the amino acid content has a greater effect than the order. Tsutsumi *et al*. investigated the role of the negatively charged linker from the molecular chaperone, heat shock protein 90 (Hsp90) ATPase [29]. They found that replacing the negatively charged linker with uncharged sequences of equivalent or longer lengths results in a structural disruption that decreases the ATPase activity. While there is variability in linker lengths and sequences from several native Hsp90 proteins, the examined native linkers all have a net negative charge. The conservation in negatively charged linker sequences additionally highlights the importance of the amino acid content in linker function.

In the work presented here, we investigate linkers from cellulase enzymes. Cellulases are often found as multi-modular enzymes with CBMs connected to GH domains. The CBM serves to increase the local concentration of enzyme near the substrate [30], [31], and the GH catalytic domain is responsible for hydrolysis of cellulose (or hemicellulose) to soluble sugars [32]. The GH and CBM domains are known to work synergistically, and separation of the two domains via proteolytic cleavage of the linker typically results in a reduction in the concentration of catalytically engaged GH domains on cellulose, and thus an overall reduction in carbohydrate turnover [33], [34]. A number of studies have examined the effects of altering linker lengths and sequences on the rate of hydrolysis for GH enzymes, demonstrating that modified linkers can affect enzyme binding capacity as well as activity [8], [35], [36]. While the results highlight the importance of the linker region for these enzymes, the functional role of cellulase linkers is not yet fully understood.

Numerous studies have focused on the flexibility and function of GH Family 6 and 7 linkers. Small-angle X-ray scattering has been used to investigate the flexibility of a linker from a GH Family 7 protein (*Trichoderma reesei* Cel7A) and a GH Family 6 protein (*Trichoderma reesei* Cel6A). Both studies found that the GH Family 6 and 7 linkers from *Trichoderma reesei* adopt extended conformations [18], [20], [37]. Additionally, cellulase linkers are often extensively *O*-glycosylated [38]–[41]. While the role of glycosylation of cellulase linkers is not entirely clear [42], *O*-glycans have been shown to confer protease resistance, and have been hypothesized to add rigidity [35], [43]. Work from our group put forth an alternative hypothesis validated by molecular dynamics (MD) simulation that the *O*-glycans serve not to rigidify, but rather extend the distance of the linker, and thus the operating distance for the enzyme by excluded volume effects [44]. Ting *et al.* propose the CBM and GH movements are synergistic, so that the two domains work together to pull a polysaccharide chain from cellulose [45]. The correct amount of rigidity of the linker, based on their hypothesis, is key to the coupling of the CBM and GH movements. Igarashi *et al.*, using high-speed atomic force microscopy, saw no difference in the rate of movement of intact *T. reesei* Cel7A and catalytic domain alone, suggesting the CBM is not required for processive movement on crystalline cellulose [46]. Thus, results from these various studies are generally not in agreement, and the behavior and function of cellulase linkers is not fully understood.

**Figure 1:**
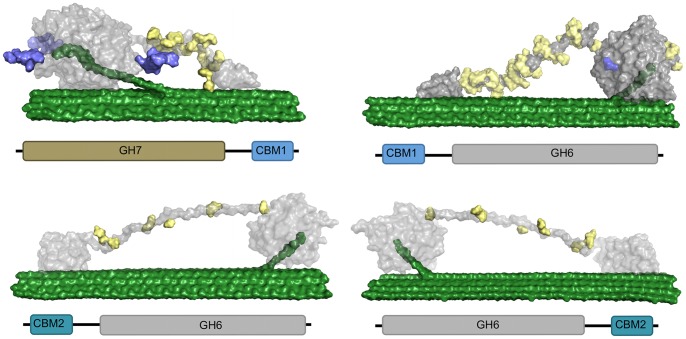
Domain order for GH Family 6 and 7 enzymes containing Family 1 or 2 CBMs. GH Family 7 enzymes are not found in bacteria. CBM Family 1 domains are found almost exclusively in fungi, and CBM Family 2 domains are often found in bacterial enzymes. The eukaryotic sequences for GH Family 6 or 7 connected to CBM Family1 are referred to as GH7/CBM1 or CBM1/GH6. The bacterial sequences for GH Family 6 bound to CBM Family 2 are referred to as GH6/CBM2 or CBM2/GH6, based on relative domain orientation.

Here we compare linkers from 4 data sets as illustrated in Figure 1:

Eukaryotic cellulases with GH Family 7 catalytic domains connected to Family 1 CBMs,
Eukaryotic cellulases with GH Family 6 catalytic domains connected to Family 1 CBMs,
Bacterial cellulases with GH Family 6 catalytic domains and Family 2 CBMs wherein the CBM is located at the N-terminus,
Bacterial cellulases with GH Family 6 catalytic domains and Family 2 CBMs wherein the catalytic domain is positioned at the N-terminus.

These data sets were chosen because GH 6 and GH 7 cellulases are among the most industrially relevant cellulases for biofuels applications [47], [48], because these enzymes and CBMs are relatively well characterized in terms of function [49]–[55], and because these data sets enable comparisons between GH Families, between domain order, and between bacterial and fungal cellulases.

The primary questions that we examine in this study are (1) whether the cellulase linkers exhibit conservation of length or (2) conservation of amino acid content, and (3) whether there is conservation in the amount and distribution of linker glycosylation. Conservation of linker characteristics implies the presence of a functional role for linkers. The comparison of linker sequences from the aforementioned sets allows us to investigate trends in linker characteristics for cellulase enzymes from different organisms, with domain substitutions and with different domain order.

We find that trends in linker length are different when different cellulase domains are present, suggesting that length is optimized based on the type of cellulase domains. MD simulations of a select set of linkers show that *O*-glycans serve to extend the linker peptides. Interestingly, we find the proline content of the bacterial linkers is more than double that of the eukaryotic linkers. High proline content can lead to extended peptide conformations [56], [57], thus bacterial linkers may have an additional route to achieve extended distances between structured domains. The serine and threonine residues are on average evenly distributed across all linker sets, where extensive *O*-linked glycosylation can occur, with the exception that there are fewer serines and threonines and more glycines at the linker termini. However, very few putative *N*-glycan sites are observed. Overall, this studies suggests that cellulase linkers have several conserved characteristics that suggest that linkers exhibit function beyond simple domain connectivity.

## Results

GHs are subdivided into families based on sequence similarity [58]. Two important cellulase families, GH Family 6 and 7, constitute the major components of industrial enzyme cocktails and thus have thus been the focus of considerable protein engineering efforts [47], [48], [59]–[61]. GH Family 6 is found in eukaryotes and bacteria, although to date GH Family 7 is found almost exclusively in fungi with the recent discovery of GH7s in a few animals [62], [63]. Secreted GHs are often connected to CBMs. Family 1 and 2 CBMs are included in this study, as they have both been shown to bind to the hydrophobic face of cellulose [64]–[66]. CBM Family 1 is found almost exclusively in eukaryotes, while CBM Family 2 is more prevalent in bacteria. By including proteins with GH Family 6 or 7 connected to CBM Family 1 or 2, we obtain the following domain combinations, with domains listed as they occur from N- to C-terminus: from eukaryotes, GH6 connected to CBM1 (CBM1/GH6) and GH7 connected to CBM1 (GH7/CBM1), and from bacteria, GH6 connected to CBM2 (CBM2/GH6), and with the domains in reverse order, (GH6/CBM2). Protein sequences from each of the four datasets shown in Figure 1 were separated into individual domains: CBM, GH or linker. Organisms and GenBank accession numbers for the proteins in each dataset are listed in Information S1 (Tables SA–SD).

There is significant divergence of linker lengths within two of the examined datasets. The eukaryotic GH6/CBM1 linkers range from almost no linker up to 129 residues, with three linkers that are more than 100 residues in length (Figure S1A). The bacterial CBM2/GH6 linker set ranges from 14 residues to 158 residues, with five linkers that are more than 100 residues in length (Figure S1B). Importantly, the eukaryotic GH6/CBM1 linkers greater than 100 residues in length are from a group of enzymes from ruminal fungi (Information S1, Table SE). While there is significant overlap in the genera represented in the eukaryotic GH7/CBM1 and CBM1/GH6 datasets, the ruminal fungi are only seen in the GH6/CBM1 set of proteins, and account for all of the linkers with greater than 100 residues. Similarly, the bacterial CBM2/GH6 linkers greater than 100 residues in length come from proteobacteria, and while there is significant overlap in the genera represented in both bacterial datasets, the proteobacteria are only seen in the CBM2/GH6 set of proteins and account for all of the linkers with length greater than 100 residues (Information S1, Table SF). Phylogenetic trees of the full-length proteins from the eukaryotic CBM1/GH6 and the bacterial CBM2/GH6 show the ruminal fungi and the proteobacteria on branches separated from the remainder of the respective datasets (Figures S2 and S3 respectively). These linker sequences are divergent in amino acid content as well length (Figure S4) and were therefore removed from the respective linker datasets compared in the subsequent sections.

### Different Cellulase Domains have Different Linker Lengths

**Figure 2:**
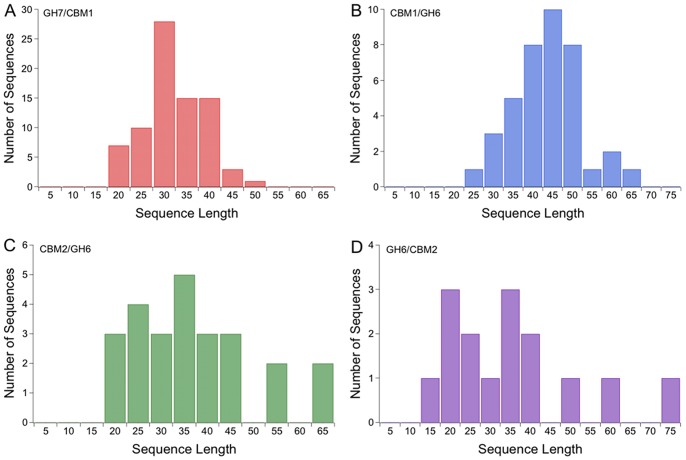
Linker sequences are divergent in length. The number of sequences for given lengths are shown for the (A) eukaryotic GH7/CBM1, (B) the eukaryotic CBM1/GH6, and the bacterial GH Family 6 datasets (C) CBM2/GH6 and (D) GH6/CBM2. Linker lengths are binned into five residues groups.

We investigated whether linker length is a conserved feature and thus important for cellulase function by comparing the average and median linker lengths for each data set. Results in Table 1 and Figure 2 suggest that linkers connecting cellulase domains from different families exhibit different average lengths, and a Student’s t-test confirms that these values are statistically significantly different at a 95% confidence level (Information S1, Table SG). The eukaryotic linkers connect a Family 1 CBM and either a Family 6 or Family 7 GH. Yet the GH7/CBM1 linkers have an average length of 30, while the eukaryotic CBM1/GH6 linkers have an average length of 42 (Table 1 and Figure 2). The distribution of linker lengths is shifted as well, with GH7/CBM1 having shorter maximum and minimum linker lengths compared to CBM1/GH6 linkers. While this difference in average linker lengths could result from the comparison of proteins from different species, there is significant overlap of genera between the two datasets; 82% of the genera in the GH7/CBM1 dataset are present in the CBM1/GH6 dataset, and 62% of the genera in the CBM1/GH6 dataset are present in the GH7/CBM1 dataset. Thus the observed differences in linker characteristics do not appear to be the result of a bias in the cellulases that have been sequenced.

**Table 1:** Lengths are compared for eukaryotic GH7/CBM1 and CBM1/GH6 and bacterial CBM2/GH6 and GH6/CBM2 linkers.

	Average	Median	Max	Min	Range	Standard deviation	Total # of Sequences
**Eukarya GH7/CBM1**	30	29	47	16	31	7	79
**Eukarya CBM1/GH6**	42	43	61	25	36	8	39
**Bacteria CBM2/GH6**	35	34	65	16	49	13	25
**Bacteria GH6/CBM2**	34	31	75	14	60	17	15

The eukaryotic CBM1/GH6 linkers have a longer average and median length compared to the eukaryotic GH7/CBM1 linkers. The bacterial CBM2/GH6 and GH6/CBM2 linkers have shorter average and median lengths compared to the eukaryotic CBM1/GH6, but have a longer range of linker lengths than either set of eukaryotic linkers.

The average length for CBM2/GH6, at 35, is similar to the average length for GH6/CBM2, at 34, suggesting domain order does not influence linker length. In addition, the minimum linker length of 16 for CBM2/GH6 is close to the minimum length of 14 for GH6/CBM2. The range for the linker lengths appears to be quite different, although this difference results from a single linker in GH6/CBM2 that is 75 amino acids long, compared to the longest linker from CBM2/GH6, which is 65 amino acids. Thus the average linker lengths for both bacterial linker sets are similar, but there are only 25 and 15 sequences in the CBM2/GH6 and GH6/CBM2 datasets respectively. Similar overlap of genera is seen for the bacterial CBM2/GH6 and GH6/CBM2 datasets as seen in the eukaryotic linkers. A Student’s t-test indicates that the linker length data for the two bacterial datasets, CBM2-GH6 and GH6-CBM2, are not statistically significantly different (Information S1, Table SG). The eukaryotic CBM1-GH6 linker lengths are statistically significantly different from the bacterial CBM2-GH6 length lengths, but not significantly different from the bacterial GH6-CBM2 length lengths despite the fact that the average and median values are nearly identical for both bacterial linker datasets. However because the GH6-CBM2 set of sequences has only 15 members, it is difficult to draw definitive conclusions from this smaller data set.

### Linker Sequences have Low Sequence Conservation, but Show Similarity in Amino Acid Content

**Figure 3:**
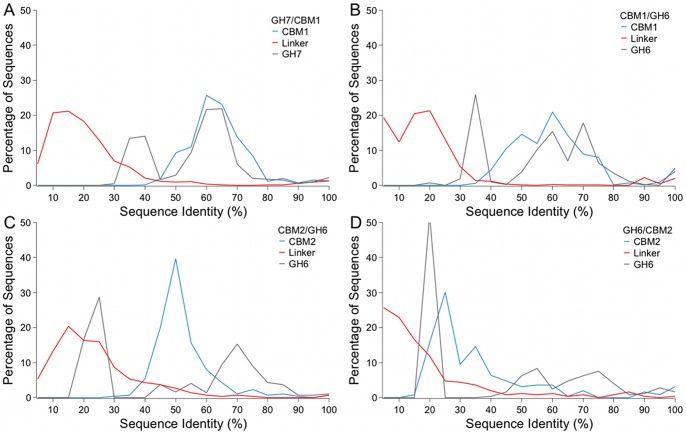
GH Family 6 and 7 linkers show lower sequence conservation compared to the structured domains. The percent sequence identity was computed from the sequence alignments for the CBM (blue), linker (red) and catalytic domain (gray) of the (A) eukaryotic GH Family 7-CBM Family 1, (B) the eukaryotic GH Family 6-CBM Family 1, and the bacterial GH Family 6-CBM Family 2 datasets (C) with the CBM located at the N-terminus and (D) with the CBM located at the C-terminus.

Linkers are generally unstructured regions that can be identified by IDP hallmarks, including low sequence conservation and bias in amino acid content [25], [67]. The linkers in all four datasets investigated here have lower sequence conservation compared to that of the structured domains, as determined by sequence identity (Figure 3). The structured domains contain some diversity, which is apparent in the sequence identity profiles. For example, the GH7 proteins are well annotated, showing that within this set of homologous proteins are both exoglucanases (processive enzymes), and endoglucanases (non-processive enzymes). As a result, we see two separate peaks in the sequence identity graph for the GH7 domain. The peak with higher sequence identity representing the comparison of endoglucanases to endoglucanases and exoglucanases to exoglucanases, and the peak with lower sequence identity results from the comparison of endoglucanases to exoglucanases (Figure 3A). Separating the GH7 catalytic domains into endoglucanases and exoglucanases results in an increase in sequence identity (Figure S5). Still, the peaks with lower sequence identity from the profiles for structured domains show higher levels of conservation than is seen in the linker sequences. Thus, despite the presence of sub-families resulting in a lower apparent sequence identity, the sequence identity for the structured domains is still clearly higher than that of the linkers.

**Figure 4:**
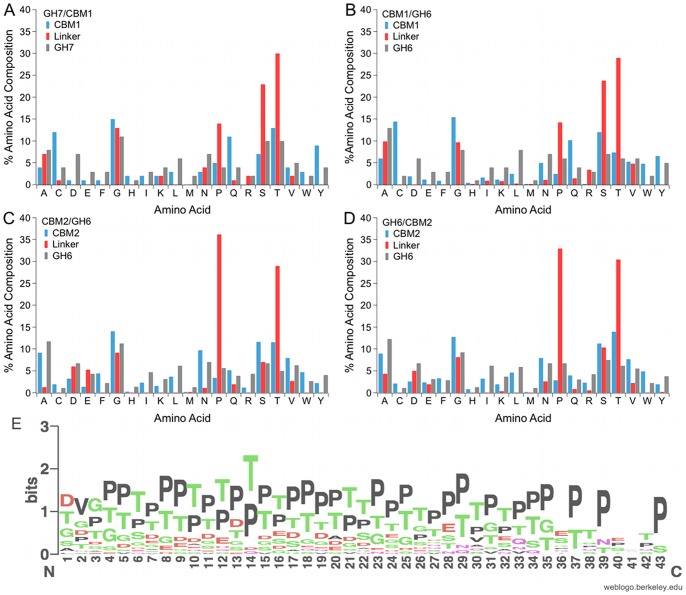
Amino acid composition for linkers shows an enrichment in serine, threonine and proline residues. (A) eukaryotic GH7/CBM1, (B) eukaryotic CBM1/GH6, (C) bacteria CBM2/GH6, and (D) bacteria GH6/CBM2. (E) A LOGO [68] for the linker regions from the bacterial CBM2/GH6 show low complexity resulting from the amino acid bias.

We also see amino acid bias in all four sets of linker sequences, with high enrichment in serine, threonine and proline, as has been noted in previous work (Figure 4A, 4B, 4C and 4D) [35]. Strong amino acid bias leads to sequences with low sequence complexity, as is apparent in the LOGO [68] representation for the bacterial CBM2/GH6 linkers (Figure 4E). However, amino acid content is conserved. Minus the ruminal fungi, the eukaryotic linker datasets, GH7/CBM1 and GH6/CBM1, have a nearly identical amino acid content, with approximately 55% combined serine and threonine, and a high proline content of approximately 15%. The two sets of bacterial linkers, CBM2/GH6 and GH6/CBM2, minus the proteobacterial sequences, have a similar amino acid content, but quite different than the eukaryotic datasets. The proline content in both the bacterial datasets, for example, is approximately 35%, but only 15% in the eukaryotic datasets. The threonine content is similar in all datasets, but the serine content is much higher in the eukaryotic sequences compared to the bacterial sequences, with approximately 25% for the eukaryotic linkers compared to 10% for the bacterial linkers (Figure 4). While it is not clear why the bacteria have fewer serine residues per linker, the result is fewer putative *O*-glycan sites. These results suggest that linkers can retain some degree of protease resistance with fewer *O*-glycans. We note that all bacterial organisms included in this work exhibit glycosyltransferases, based on the presence of genes listed in CAZY.

**Figure 5:**
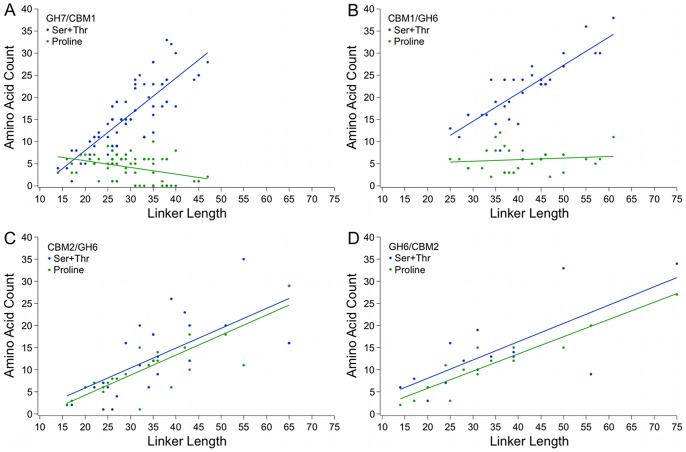
Serine and threonine content increases with length, but proline content increases only in bacterial linkers. The proline content does not increase with the serine and threonine content in the (A) eukaryotic CBM1/GH7 linkers or the (B) eukaryotic GH6/CBM1 linkers, but proline content increases with serine and threonine content for the (C) bacterial CBM2/GH6 and (D) GH6/CBM2 linkers. R^2^ values for trend lines reported in Information S1, Table SH.

As shown in Figure 5, the combined serine and threonine content increases as linker lengths increase for both eukaryotic and bacterial linkers. The proline content increases with linker length for the bacterial linkers, similar to the trend seen for serine and threonine content. There is no increase in proline content for the eukaryotic linkers, however, regardless of linker length (Figure 5). The higher proline content in the bacterial linkers is therefore not the result of a few sequences with higher proline content, but instead demonstrates an apparent trend.

Both the eukaryotic GH6/CBM1 dataset and the bacterial CBM2/GH6 dataset contained a subset of sequences from organisms not seen in their counterpart dataset, and which had some linker regions with highly divergent linker lengths. The amino acid content for these subsets was analyzed separately, and is highly divergent just as with the linker lengths. The eukaryotic GH6/CBM1 linkers from ruminal fungi not only contain some significantly longer sequences, but the amino acid content is unlike any other linkers evaluated in this set of proteins. The linkers for this dataset are approximately 45% asparagine, with just 5% combined serine and threonine content (Figure S4A). (Thus these linker sequences are nearly devoid in putative glycosylation sites.) The proteobacterial CBM2/GH6 linkers have the highest glycine content seen in any of the linker sets examined here, with approximately 25% glycine, almost double the glycine content seen in any of the other linker datasets. The combined serine/threonine content is similar to that seen in the other datasets, but the distribution is quite different, with approximately 50% serine and only 10% threonine (Figure S4B). Thus the amino acid content seems to be conserved regardless of domain substitution, as with the eukaryotic GH7 and GH6 linkers, or reverse the order of the domains, as with the bacterial CBM2/GH6 and GH6/CBM2 linkers.

### Glycosylation May Play Multiple Roles in Linker Sequences

**Figure 6:**
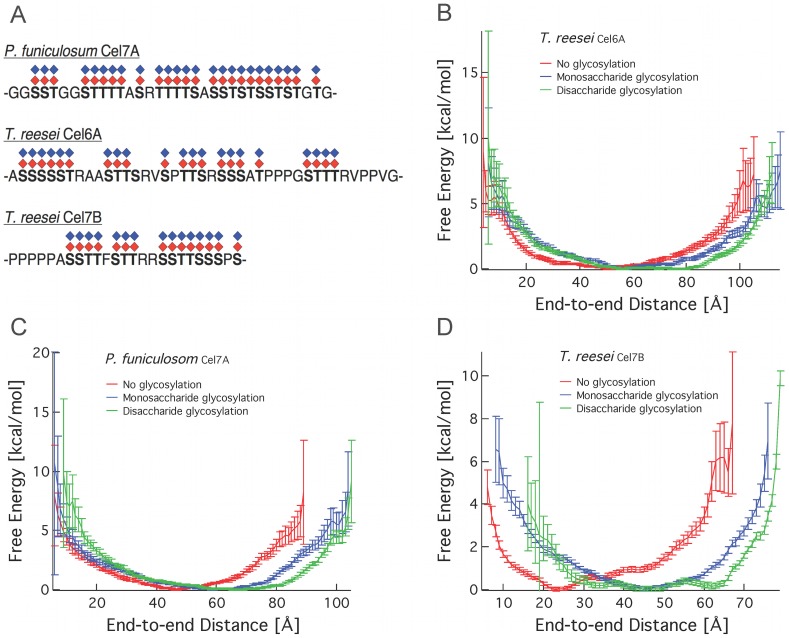
Glycosylation extends end-to-end distance of linkers. Relative free energy as a function of end-to-end distance from the REMD simulations for linkers. (A) The linker sequences examined in this study, shown with the putative *O*-glycosylation sites. REMD simulations were conducted with no glycans, a monosaccharide at each glycan site, and a disaccharide at each glycan site. (B) Free energy as a function of end-to-end distance for the *T. reesei* Cel6A linker. (C) Free energy as a function of end-to-end distance for the *T. reesei* Cel7B linker. (D) Free energy as a function of end-to-end distance for the *Penicillium funiculosom* Cel7A linker.

To gain further insights into the physical role of glycans on linkers, we evaluated three linkers using circular dichroism and MD simulations. We assessed the level of secondary structural elements of the linkers by performing circular dichroism (CD) on three non-glycosylated linker peptides, in addition to the linker peptide examined by our group in previous work [44]. All four peptides are largely unstructured, as indicated by the presence of a minimum in molar elipticity at approximately 200 nm (Figure S6). The CD spectra for all four peptides are indicative of random coil conformations. We evaluated the flexibility and movement of the four peptides using MD simulations, comparing the free energy as a function of end-to-end distance for linkers with and without *O*-mannose residues. Expression host and growth conditions can affect glycosylation patterns, making the determination of a glycosylation pattern for a given protein difficult [39], [41], [69]–[73]. For this reason, we investigate linkers with no glycosylation, with a monosaccharide, and with a disaccharide attached to each serine and threonine residue. This work is an extension of previous work with the *T. reesei* Cel7A linker (Figure 6A) [44]. We see that the linkers are not rigidified by the addition of either mono- or disaccharides at each serine or threonine based on the end-to-end distance distributions of the linker peptides. The linkers are extended incrementally, however, when comparing the addition of zero, one or two glycans at each serine or threonine, as measured by shifts in the most probable end-to-end distance of the linker peptides (Figure 6).

**Figure 7:**
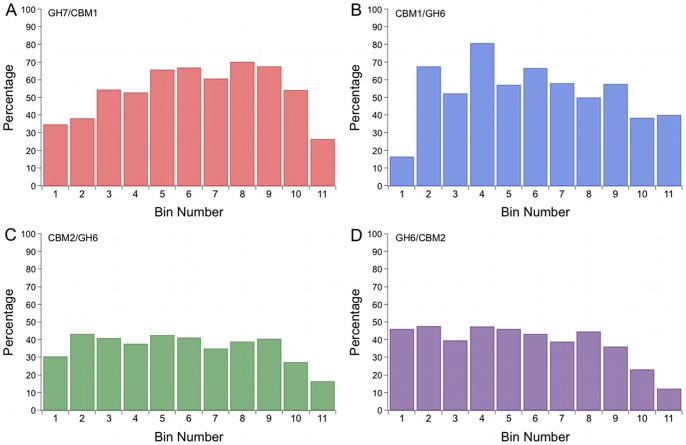
*O*-linked glycosylation sites distributed evenly across the linker regions. The probability of finding serine or threonine residues was computed for sections of the linker sequences for (A) the eukaryotic GH Family 7, (B) the eukaryotic GH Family 6, and the bacterial GH Family 6 datasets with (C) the CBM located at the N-terminus and (D) with the CBM located at the C-terminus. Each sequence was split into 11 approximately equal sections, or bins, from N- to C-terminus. The number of combined serine and threonine residues was divided by the number of sequence positions for each bin.

**Figure 8:**
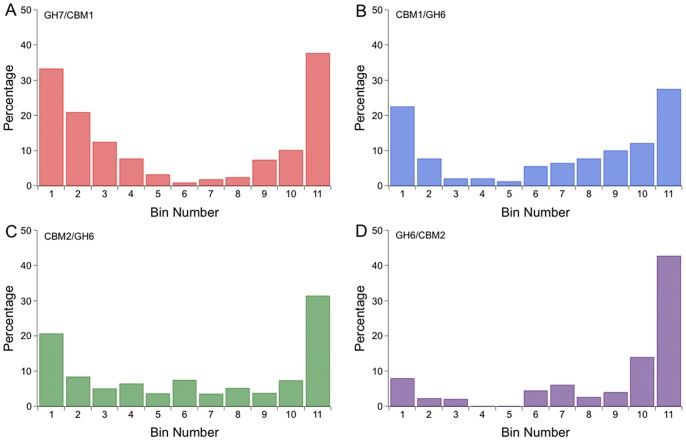
An increased occurrence of glycine residues occurs where the linkers connect to globular domains. The probability of finding a glycine residue was determined for the linker sequences in (A) the Eukaryotic GH Family 7, (B) the Eukaryotic GH Family 6, and the Bacterial GH Family 6 datasets (C) with the CBM located at the N-terminus and (D) with the CBM located at the C-terminus. Each sequence was split into 11 sections, or bins, with an approximately equal number of residues in each bin. The number of glycine residues was divided by the total number of sequence positions in each bin.

The location of putative *O*-glycan sites is also informative, whether for protease resistance, for linker extension, or for other functional reasons. We investigate the distribution and amount of putative *O*-glycan sites by determining the percent serine/threonine content along the length of the linker sequences. We start by aligning the linker sequence in each dataset and dividing each sequence into eleven bins, each bin containing approximately the same number of amino acids. Thus an 11 amino acid sequence would have one residue per bin, a 22 amino acid sequence would have two residues per bin, etc. Finally, the number of combined serine and threonine residues was divided by the total number of residues in that bin. The results in Figure 7 suggest that the putative *O*-glycan sites are, on average, evenly distributed across the linker sequences for all four datasets considered. The percentage of putative *O*-glycan sites is slightly lower, however, near the linker termini where the linker connects to a structured domain. Further, we examined the glycine content along the length of the linker sequences. The results show that there is a significantly higher glycine content at the linker termini, where the linkers connect to structured domains (Figure 8). The high occurrence of glycine residues at the junctions between the structured domains and the linkers likely imparts additional flexibility, and may play a role in domain orientation when the enzymes are bound to the substrate. These results may indicate the importance of flexibility over extension in the regions where structured domains connect to linker peptides to allow for correct domain orientation on the substrate.

High proline content can lead to extended peptide conformations [57]. We therefore evaluated the proline content for all four sets of linker sequences as well. The bacterial linkers exhibit twice the proline content of the eukaryotic linkers, with approximately 35% and 15% respectively. At the same time, putative *O*-glycosylation sites are decreased for bacterial linkers compared to eukaryotic linkers as threonine content is approximately equal for all linker sets, with approximately 30%, but serine content for the bacterial linkers is less than half that of the eukaryotic sequences, with approximately 10% and 25% respectively (Figure 4). Thus there may be multiple mechanisms that a protein can use to achieve extended conformations. The distribution of proline residues across linkers was determined using the same method used to assess *O*-glycan distribution, described above. Proline residues are evenly distributed across the linkers (Figure S7).

Putative *N*-glycan sites display a markedly different pattern than putative *O*-glycan sites. While *O*-glycans may be attached to any serine or threonine residue, a specific amino acid sequence must be present for the attachment of an *N*-glycan, which is N-X-S/T where X can be any amino acid other than proline. For all the asparagines in the combined set of linker sequences (minus the asparagine-rich linkers from the ruminal fungi), 150 in all, there are only 7 *N*-glycan sequons (Table 2). Additionally, there are 100 asparagines followed immediately by prolines, thus virtually precluding the possibility of the attachment of an *N*-glycan. The absence of *N*-glycans in cellulase linkers could be due to many things, including the presence of glycan-trimming enzymes [71] or the role *N*-linked glycosylation plays in protein folding and stability [74]. These results suggest *N*-glycosylation is evolutionarily avoided in GH linker regions.

**Table 2:** There is a bias against N-linked glycosylation sequons for examined linker regions.

	GH7/CBM1					CBM1/GH6					CBM2/GH6						GH6/CBM2						
**N-X-S/T (X≠P)**	5					0					1						1						
**N-P**	68					5					5						22						
**Total N’s**	91					18					10						31						
**Total P’s**	334					275					316						204						
**Total # Residues**	2361					2170					873						1213						
AAS82858.1:	P	E	P	T	**N**		**P**	T	**N**	**P**		T	**N**	**P**	T	**N**		**P**	T	**N**	**P**	G	

While a few N-linked sequons are found on the examined linker sequences, a proline residue immediately follows the majority of asparagine residues, precluding the possibility of an N-linked glycosylation site.

## Discussion

Overall, the results presented here suggest that cellulase linker lengths are optimized for specific structured domains. The eukaryotic linkers include either a Family 6 or 7 GH attached to a Family 1 CBM. The average linker length is nearly 50% greater for the GH Family 6 linker than the GH Family 7 linker, with a average lengths of 42 and 30, respectively. The bacterial linker sequences contain Family 6 GHs attached to Family 2 CBMs, with the linkers divided into two groups based on domain order. Both sets of bacterial linker sequences have a similar average linker length, with 35 for CBM2/GH6 and 34 for GH6/CBM2 (listed N- to C-terminus). Thus linker lengths may depend on the structured domains present but not on the domain order. A bias in the organisms that have been sequenced could lead to the apparent differences in length or amino acid content reported here. There is significant genera overlap, however, between the two eukaryotic linker sets (GH7/CBM1 and CBM1/GH6) and the two bacterial linker sets (CBM2/GH6 and GH6/CBM2) (Information S1, Table SI). Thus a strong sequencing bias is not apparent between the two eukaryotic or two bacterial datasets.

Related to previous experimental work wherein linker length may play a role in cellulase action, Igarashi *et al.* used high-speed atomic force microscopy to visualize crystalline cellulose degradation with the *T. reesei* GH Family 6 and 7 enzymes, Cel6A and Cel7A, respectively [75]. They observed that Cel7A is able to processively move down the length of the cellulose crystals and deconstruct cellulose via an ablative mechanism whereas Cel6A did not translate down the length of the crystals, but instead typically was typically found in the same location. It has thus been proposed that *T. reesei* Cel6A is not a processive enzyme, but rather helps to clear “traffic jams” on the cellulose surface, although the molecular-level details of why Cel7A becomes jammed are still unclear. It is conceivable that the shorter linker length in *T. reesei* Cel7A relative to *T. reesei* Cel6A is important for processivity, whereas the longer linker lengths in Cel6A enable it to search for hydrolytic sites of attack from a single position of the CBM on the cellulose surface.

The linker regions from the Family 6 and 7 GHs investigated exhibit characteristics of disordered, flexible regions. Sequence-based analysis of the linkers reveals little sequence conservation and significant amino acid bias, as has been seen in IDP sequences [25], [67]. Circular dichroism spectra for a select set of non-glycosylated linker sequences show an absence of secondary structural elements. Molecular simulations of the linker sequences indicate both non-glycosylated and glycosylated linkers are flexible.

While the linker regions bear all the hallmarks of IDPs, extension emerges as an important and conserved feature. Molecular simulations comparing non-glycosylated linkers to glycosylated linkers with monosaccharide and disaccharide glycans show incremental extension of the linker peptides with the presence of increasing amounts of glycosylation, extending previous results from our group [44]. The high serine and threonine content in nearly all linker sequences investigated here show the amount of *O*-glycosylation possible. Thus *O*-glycans may provide both protease resistance and linker extension for cellulases.

Comparing sequences from eukaryotic and bacterial linkers suggests that cellulase linker extension can be achieved through different mechanisms. High proline content, for example, can lead to extended conformations [56]. The proline content in the bacterial linkers, at around 35%, is more than double the proline content in the examined eukaryotic linkers. Conversely, while the threonine content is similar for all examined linkers, the serine content in the bacterial linkers is nearly half that seen in the eukaryotic linkers, resulting in a decrease in putative *O*-glycan sites from approximately 55% in the eukaryotic linkers to approximately 40% in the bacterial linkers. The reason for the differences in amino acid composition between bacterial and eukaryotic linkers is not clear at this point. We do know, however, that the expression host and the presence of different glycan-trimming enzymes from an organism’s secretome affect the extent of *O*-linked glycosylation for carbohydrate-active enzymes [42].

An ultimate goal in the use of cellulases is to design robust enzymes that can accommodate different environmental conditions or that can be expressed in a variety of host organisms for a broad range of biofuel production processes. While questions remain, this study elucidates several conserved characteristics in cellulase linkers, and highlights the need for consideration of linkers in cellulase engineering. Generally, the engineering of multi-modular enzymes can benefit from, and may require, the optimization of all enzyme components.

## Methods

### Creation of GH Family 6 and 7 Datasets

The CBM, linker and CD for eukaryotic GH Family 6 and 7 datasets (CBM1/GH6 and GH7/CBM1) and bacterial GH Family 6 datasets (CBM2/GH6 and GH6/CBM2) were generated by cross-referencing the GenBank accession codes for all eukaryotic Glycoside Hydrolase Family 6 or 7 entries, or bacterial Glycoside Hydrolase Family 6 entries, with the GenBank accession codes for either eukaryotic or bacterial CBM Family 1 or 2 entries from the Carbohydrate-Active EnZymes database (CAZY, www.cazy.org) [58]. The presence of a single CBM Family 1 or 2 was used as an inclusion criterion to ensure the sequence variability in the linker dataset represents allowable sequence space only for a limited set of conditions. The GenBank access numbers and organism names are listed in Supplementary Information (Information S1, Tables SA, SB, SD and SF). The GenBank accession numbers for the ruminal fungal enzymes from CBM1/GH6 are listed in Information S1, Table SC and the GenBank accession numbers for the proteobacterial enzymes from CBM2/GH6 are listed in Information S1, Table SE. The resulting sequence datasets were separated into CD, linker domain and CBM using the criteria detailed below.

The eukaryotic CBM1/GH6 members are ordered by protein domain, reading from N- to C-terminus, as CBM first, followed by the linker region and lastly the catalytic domain. For the CBM, (1) any signal peptide N-terminal to the CBM sequence was removed by deleting all sequence more than 6 residues N-terminal to the conserved QCGG motif. Then (2) the CBM was separated from the linker sequence by cutting after the conserved (S)QC(L) motif. This motif was selected based on observed atomic coordinates from a CBM1 solution NMR structure, PDB id 2cbh [30]. The sequences for the CD were isolated by cutting the sequence at five residues N-terminal to the (G)N(P)(F) motif. While this motif is not absolutely conserved, the sequence conservation is high enough to be easily identifiable in a sequence alignment using the alignment criteria described in the GH Family 6 and 7 sequence alignments and analysis section below. This motif is was selected based on observed atomic coordinates from the GH6 CD xray structure, PDB id 1qk2 [51].

The GH7/CBM1 members are ordered by protein domain, reading from N- to C-terminus, with CD first, followed by the linker region and lastly the CBM. The CD domain was isolated by separating the CD and linker sequence nine sequence positions after the conserved S/T-N/D-I-K motif. This motif was selected based on observed atomic coordinates from the GH7 CD xray structure, PDB ID 1q9h [76]. The sequences for the CBM1 domain were separated from the linker region by cutting 6 residues N-terminal to the conserved QCGG motif. This motif was selected based on observed atomic coordinates from a CBM1 solution NMR structure, PDB ID 2cbh [30].

The bacterial CBM2/GH6 members are ordered by protein domain, reading from N- to C-terminus, with CBM2 first, followed by the linker region and lastly GH6. The CBM was isolated by separating the CBM and linker sequences two residues C-terminal to the absolutely conserved cystein residue. The CD sequences contain several motifs at the N-terminus, thus the CD was isolated by cutting at either the RVDN motif or cutting N-terminal to the YVD motif.

The bacterial GH6/CBM2 members are ordered by protein domain, reading from N- to C-terminus, with GH6 first, followed by the linker region and lastly CBM2. The GH6 was isolated by separating the GH and linker sequences eight residues C-terminal to the FVML motif. The CBM2 sequences were isolated by cutting at the conserved N-terminal cystein, where a cystein or other hydrophobic amino acid (L/I/F) is observed.

### Sequence Alignments and Analysis

Sequences were aligned and analyzed using the MacVector software (MacVector, Inc., Cary, NC). Multiple sequence alignments were performed using the GONNET matrix, with a gap opening penalty of 10 and a gap extending penalty of 0.05 (Figure S8). The aligned sequences are shown in Figure S8. The percent identity is a measure of each sequence compared to every other sequence in a given alignment (Figure 3). The alignment of the bacterial CBM2/GH6 linker sequences is shown in a LOGO representation made using WebLogo (Figure 4E) [68]. Processive and non-processive GH Family 7 cellulases were separated primarily according to the Enzyme Commission number from ExplorEnz (http://www.enzyme-database.org/), and secondarily by aligned gap regions located in the catalytic loops. Percent sequence identity was then performed independently on the GH 7 processive and non-processive enzyme datasets (Figure S4). Histograms for percent identity and linker length distributions were created using StatPlus:mac [77]. Graphs were generated using IGOR Pro (WaveMetrics Inc., Lake Oswego, OR).

### Replica Exchange Molecular Simulations for Select GH Family 6 and 7 Linker Sequences

We conduct simulations in a similar manner to our previous study [73]. We apply the CHARMM force field to model the proteins with the CMAP correction [78], [79] and the C35 force field for the carbohydrates [80], [81]. We employ the Generalized Born with Molecular Volume (GBMV) implicit solvent model [82], [83] for enhanced conformational sampling. Although the GBMV model is expensive relative to other implicit solvent models, it has been shown to reproduce the underlying free energy landscapes of peptides in explicit solvent with significantly faster conformational sampling as well as reproduce protein structures from the Protein Data Bank more accurately than other implicit solvent models [84], [85]. We run all simulations in CHARMM [86]. The SHAKE algorithm was used to constrain bond distances to hydrogens [87]. In all cases, we used a 1.5 fs time step with a 21 Å cutoff for nonbonded interactions. The GBMV model parameters are the same as in reference [44].

We used the implementation of REMD described by Lin and Shell [88]. We analyze REMD convergence with the transit time and decorrelation of replicas between temperatures as described by Abraham and Gready [89] as well as with bootstrapping to measure error bars on the free energy data. Table 1 shows the system size, temperature range, and number of replicas for each scenario examined. In all cases, the number of replicas was adjusted to ensure an acceptance rate between 40 and 50%. The free energy of the system as a function of the end-to-end distance was constructed by Boltzmann inversion for each simulation set at the lowest temperature replica (300 K in all cases). Multiple clustering algorithms were applied to each scenario via the Amber Tools PTRAJ program [90] as described in reference [91] (Figure S9). More information regarding analysis methods is provided in the Supplementary Information (Information S1, Tables SJ and SK).

### Circular Dichroism

Non-glycosylated linker peptides were purchased from Pi Proteomics, LLC. (Huntsville, Alabama, USA), which were synthesized by standard solid-state peptide synthesis methods. The termini were capped in all cases so that the linkers did not exhibit charged ends, as was done in the MD simulations, and the sequences matched those used in the MD simulations. CD measurements were conducted using a Jasco J-715 spectropolarimeter with a jacketed quartz cell with a 1.0 mm path length. The cell temperature was controlled to within +/− 0.1°C by circulating 90% ethylene glycol using a Neslab R-111m water bath (NESLAB Instruments, Portsmouth, NH, U.S.A.) through the CD cell jacket. The results were expressed as mean residue ellipticity [*Θ*]_mrw_. The spectra obtained were averages of five scans. The spectra were smoothed using an internal algorithm in the Jasco software package, J-715 for Windows. Protein samples were studied in 20 mM sodium acetate buffer, pH 5.0 with 100 mM NaCl at a protein concentration of 0.3 mg/mL for the near-UV CD. Spectra of different constructs were monitored by CD in the near UV (190–260 nm) region.

## Supporting Information

Figure S1 **Distribution of linker lengths for eukaryotic CBM1/GH6 including sequences from (A) ruminal fungi and (B) bacterial GH6/CBM2 including sequences from proteobacteria.**
(PDF)

Figure S2 **Phylogenetic analysis of full-length eukaryotic GH Family 6-CBM Family 1 proteins shows the ruminal fungal proteins (inside the red box) on a separate branch than the other fungal proteins, inferring evolutionary divergence between the two groups.**
(PDF)

Figure S3 **Phylogenetic analysis of full-length bacterial GH Family 6-CBM Family 2 proteins (GH6/CBM2) shows the proteobacterial proteins (inside the red box) on a separate branch from the actinobacterial proteins, inferring an evolutionary divergence between the two groups.**
(PDF)

Figure S4 **Evolutionarily divergent eukaryotic and bacterial GH Family 6 cellulase sequences have a markedly different amino acid composition compared to the other linker sequences examined in this work.** (A) The linkers from Eukaryotic GH6/CBM1 rumenal fungi are highly enriched in asparagine residues, and nearly devoid of serine or threonine residues. (B) The linkers from GH6/CBM2 proteobacteria are highly enriched in glycine compared to other linker sets examined in this study (Figure 4).
(PDF)

Figure S5 **The GH Family 7 catalytic domain sequences contain two functionally distinct groups, processive (endoglucanases) and non-processive (exoglucanases).** The sequence identity for GH7 catalytic domain sequences is higher when the two groups are separated into (A) exoglucanases and (B) endoglucanases compared to sequence identity for the entire set of GH7 catalytic domains (Figure 3).
(PDF)

Figure S6 **CD measurements of select Eukaryotic GH Family 6 and 7 non-glycosylated linker peptides indicate that the examined linkers are largely unstructured.**
(PDF)

Figure S7 **Proline residues are distributed evenly across the linker regions.** The probability of finding proline residues was computed for sections of the linker sequences for (A) the eukaryotic GH Family 7, (B) the eukaryotic GH Family 6, and (C) the bacterial GH Family 6 datasets with the CBM Family 2 located at the N-terminus and (D) with the CBM Family 2 located at the C-terminus. Each sequence was split into 11 approximately equal sections, or bins, from N- to C-terminus. The number of proline residues in each bin was divided by the total number of sequence positions for each bin.
(PDF)

Figure S8 **Sequence alignments for each domain from the four datasets: eukaryote GH7/CBM1, CBM1/GH6 and bacterial CBM1/GH6 and GH6/CBM1.**
(PDF)

Figure S9 **Clustering metric results for the REMD simulations using the hierarchical and average linking algorithms for Cel6A, EGI, and** ***P. funiculosum*** **linkers.** The metrics are useful for determining the optimal number of structural clusters found by REMD. The results here show there are no significantly populated, distinct structural clusters in any linker set.
(PDF)

Information S1 **Supplementary methods and tables.**
(PDF)
