# Supplementary figures and images for: Cellulase Linkers Are Optimized Based on Domain Type and Function: Insights from Sequence Analysis, Biophysical Measurements, and Molecular Simulation
Source: PLoS One. 2012 Nov 6;7(11):e48615. doi: 10.1371/journal.pone.0048615 (PMC3490864; doi:10.1371/journal.pone.0048615)

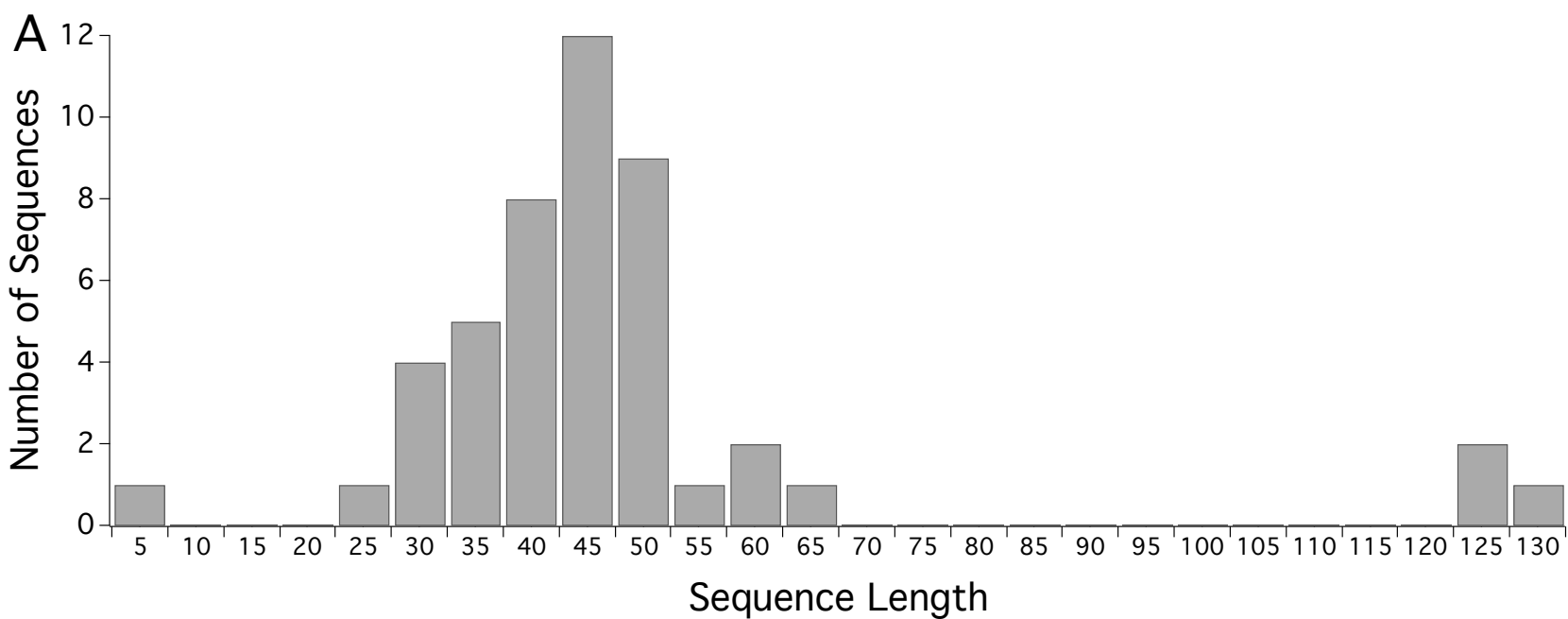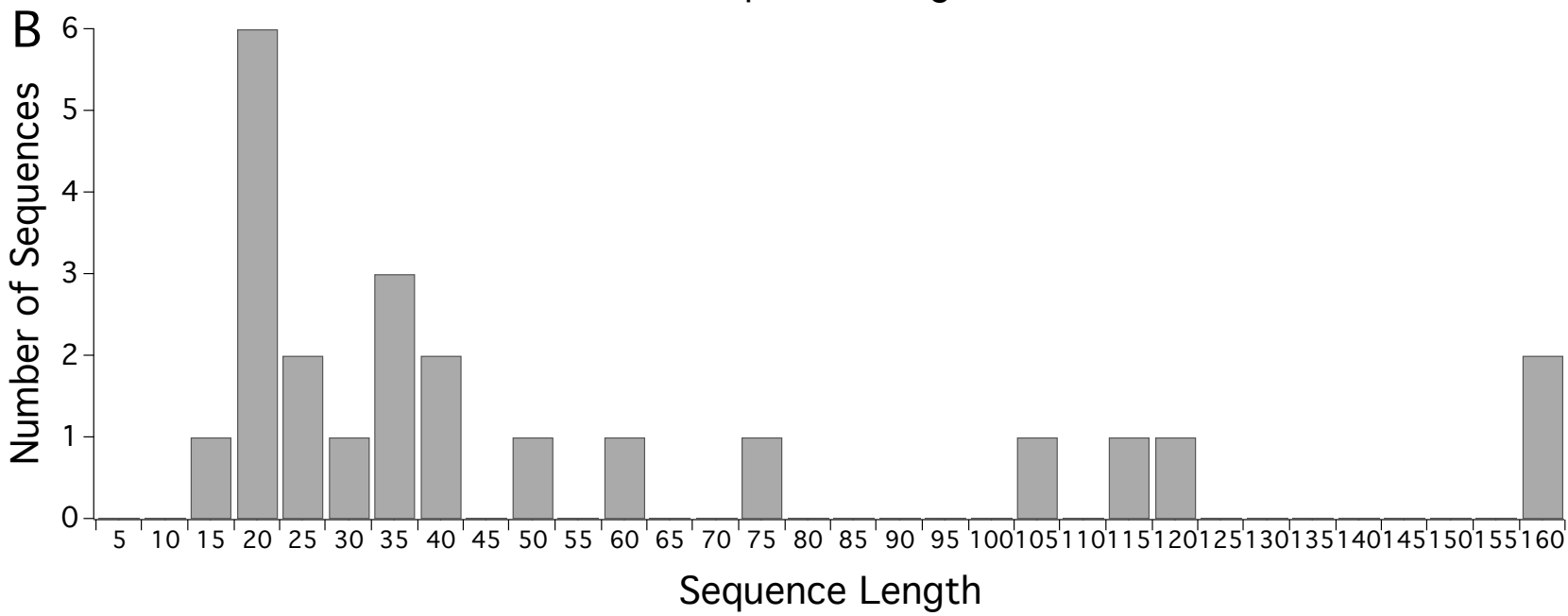

Supplement: Figure S1 — Distribution of linker lengths for eukaryotic CBM1/GH6 including sequences from (A) ruminal fungi and (B) bacterial GH6/CBM2 including sequences from proteobacteria. (PDF) [file pone.0048615.s001.pdf]

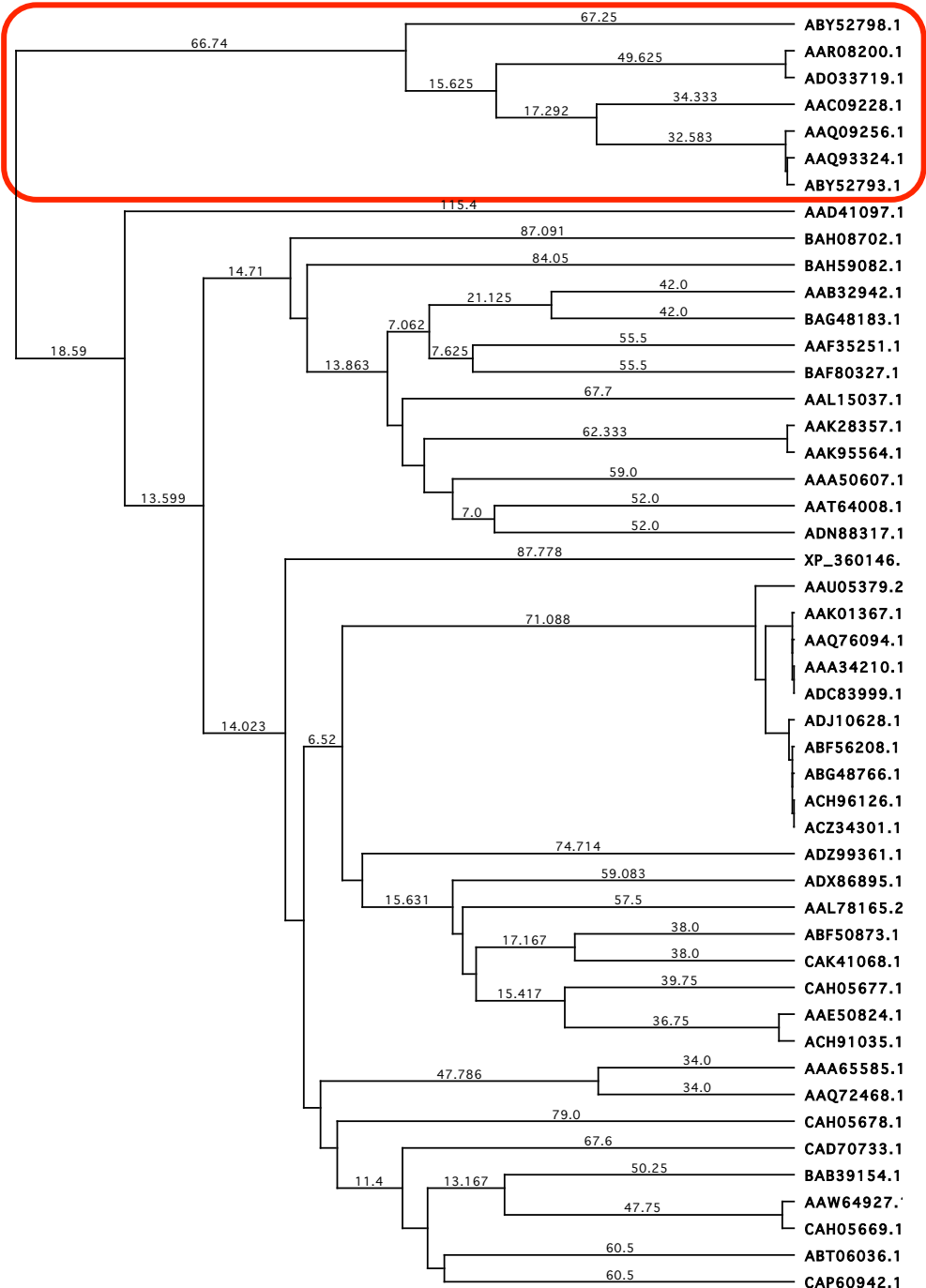

Supplement: Figure S2 — Phylogenetic analysis of full-length eukaryotic GH Family 6-CBM Family 1 proteins shows the ruminal fungal proteins (inside the red box) on a separate branch than the other fungal proteins, inferring evolutionary divergence between the two groups. (PDF) [file pone.0048615.s002.pdf]

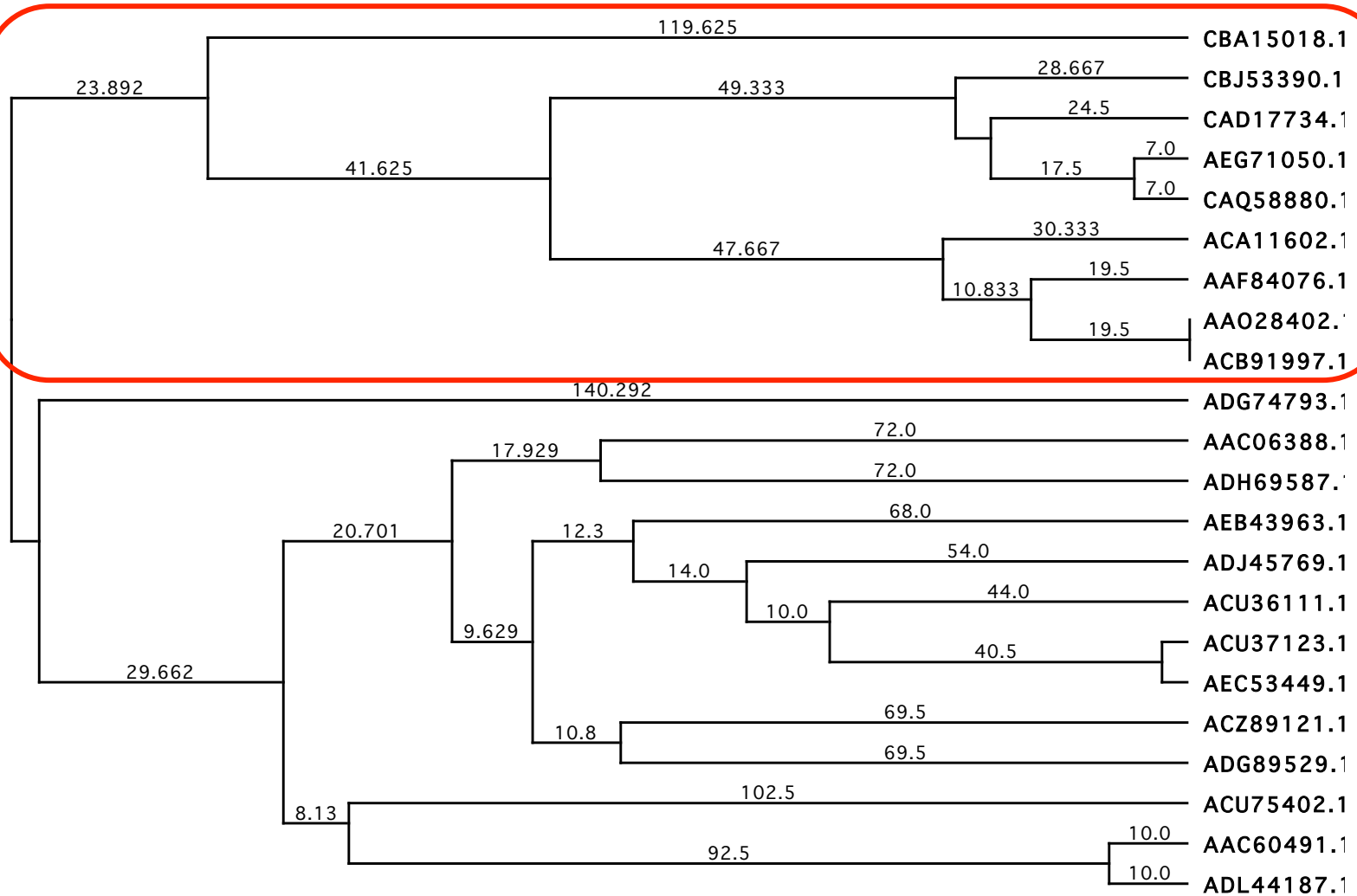

20.0

Supplement: Figure S3 — Phylogenetic analysis of full-length bacterial GH Family 6-CBM Family 2 proteins (GH6/CBM2) shows the proteobacterial proteins (inside the red box) on a separate branch from the actinobacterial proteins, inferring an evolutionary divergence between the two groups. (PDF) [file pone.0048615.s003.pdf]

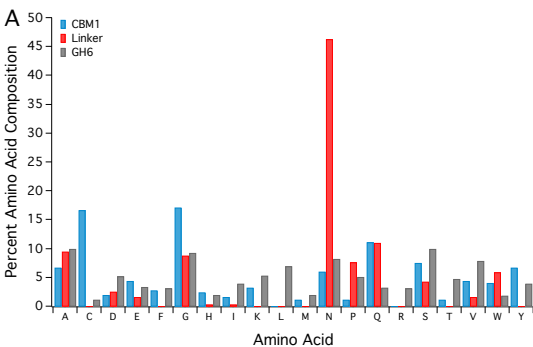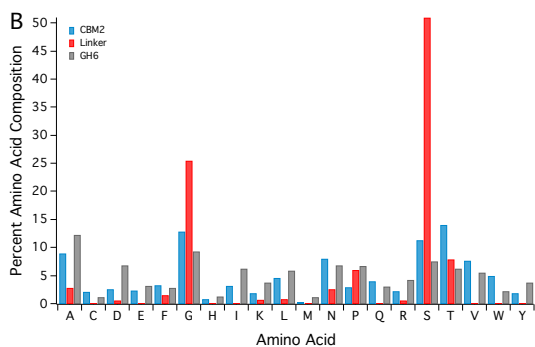

Supplement: Figure S4 — Evolutionarily divergent eukaryotic and bacterial GH Family 6 cellulase sequences have a markedly different amino acid composition compared to the other linker sequences examined in this work. (A) The linkers from Eukaryotic GH6/CBM1 rumenal fungi are highly enriched in asparagine residues, and nearly devoid of serine or threonine residues. (B) The linkers from GH6/CBM2 proteobacteria are highly enriched in glycine compared to other linker sets examined in this study (Figure 4). (PDF) [file pone.0048615.s004.pdf]

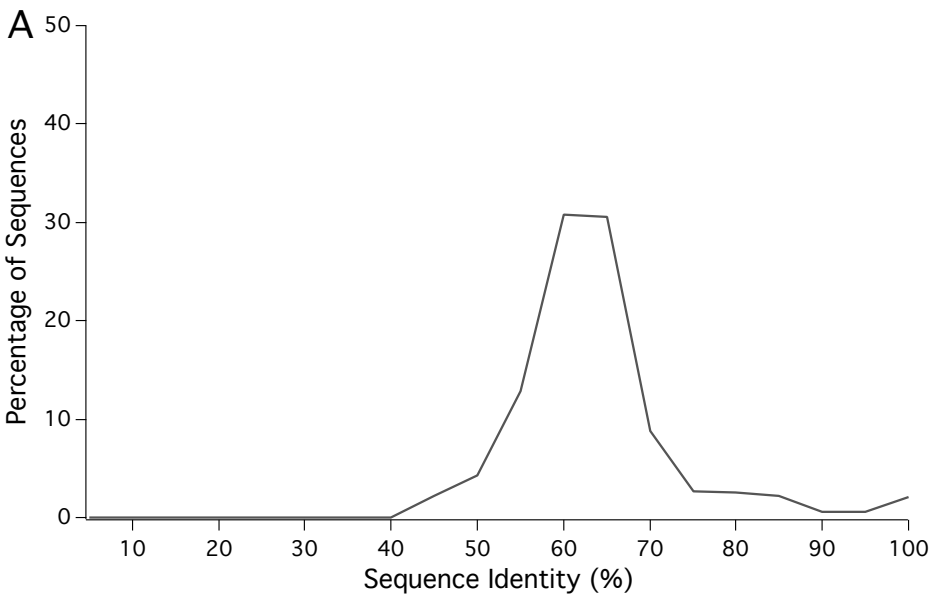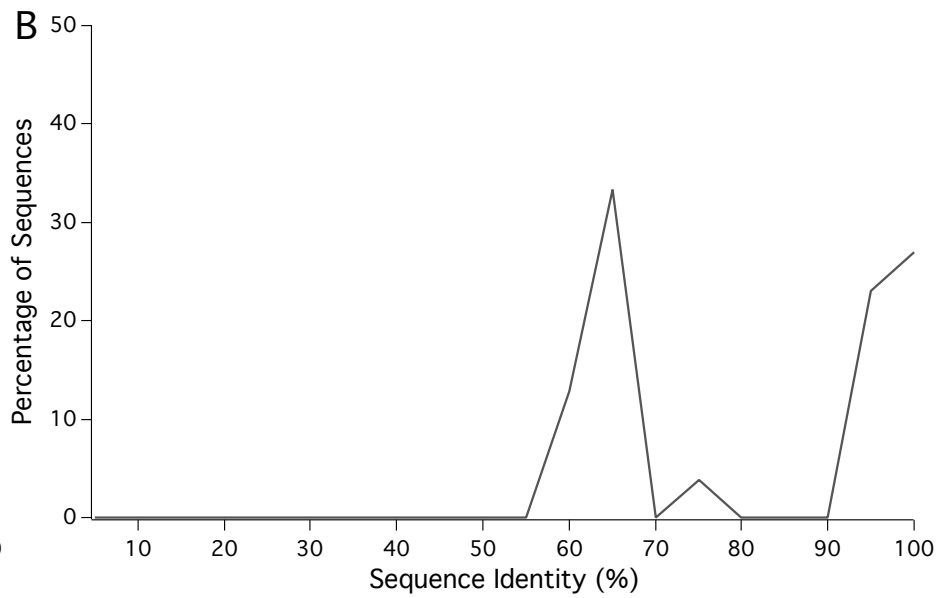

Supplement: Figure S5 — The GH Family 7 catalytic domain sequences contain two functionally distinct groups, processive (endoglucanases) and non-processive (exoglucanases). The sequence identity for GH7 catalytic domain sequences is higher when the two groups are separated into (A) exoglucanases and (B) endoglucanases compared to sequence identity for the entire set of GH7 catalytic domains (Figure 3). (PDF) [file pone.0048615.s005.pdf]

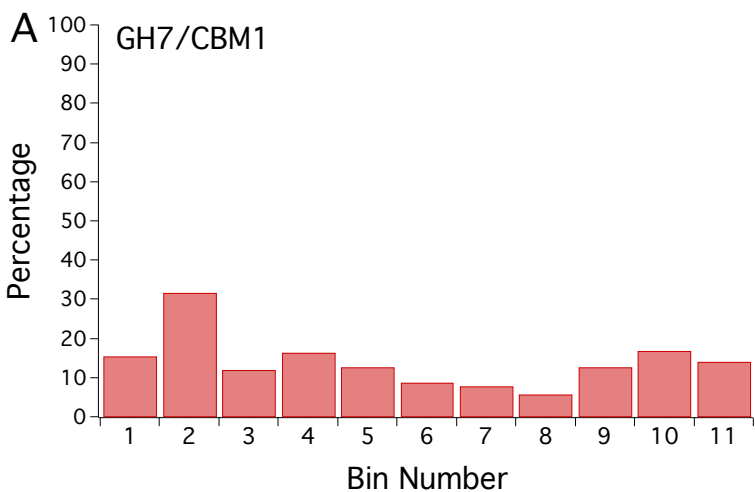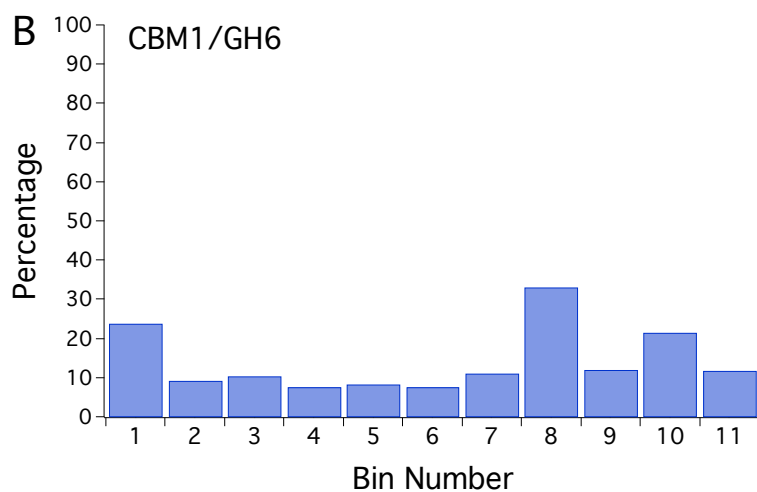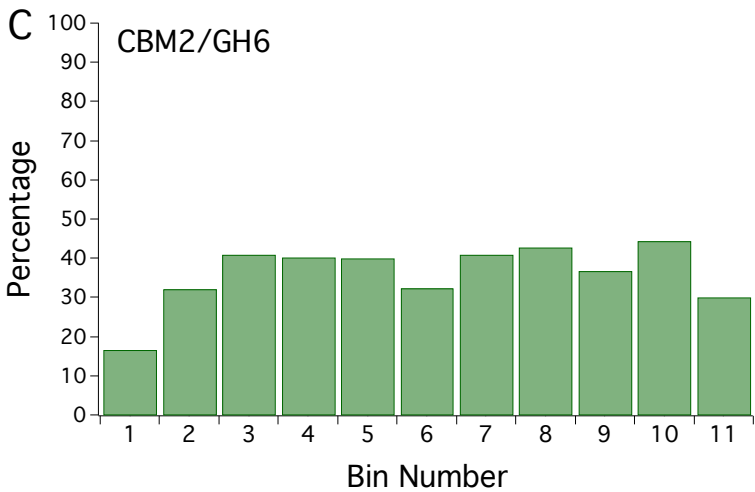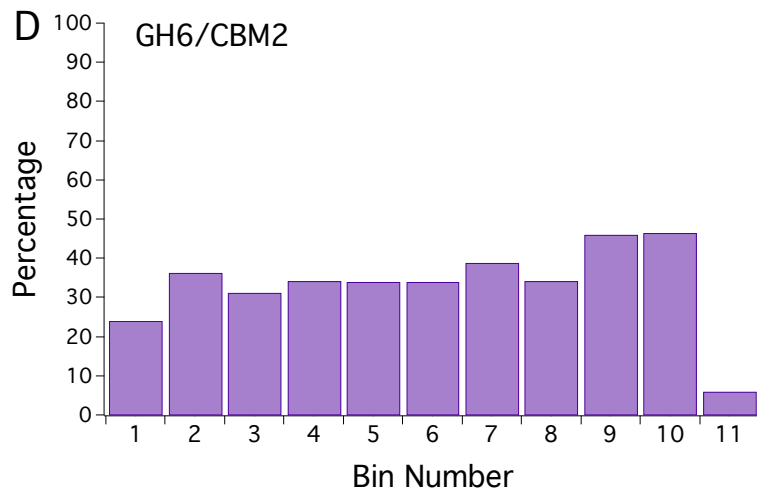

Supplement: Figure S6 — CD measurements of select Eukaryotic GH Family 6 and 7 non-glycosylated linker peptides indicate that the examined linkers are largely unstructured. (PDF) [file pone.0048615.s006.pdf]

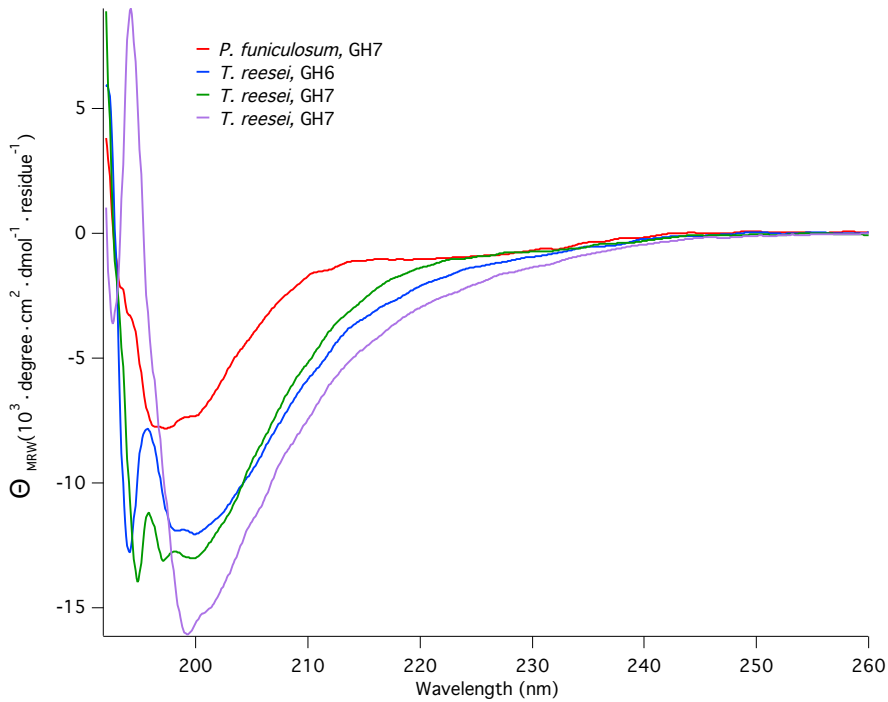

Supplement: Figure S7 — Proline residues are distributed evenly across the linker regions. The probability of finding proline residues was computed for sections of the linker sequences for (A) the eukaryotic GH Family 7, (B) the eukaryotic GH Family 6, and (C) the bacterial GH Family 6 datasets with the CBM Family 2 located at the N-terminus and (D) with the CBM Family 2 located at the C-terminus. Each sequence was split into 11 approximately equal sections, or bins, from N- to C-terminus. The number of proline residues in each bin was divided by the total number of sequence positions for each bin. (PDF) [file pone.0048615.s007.pdf]

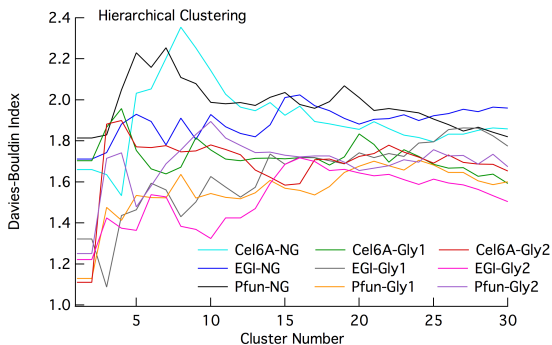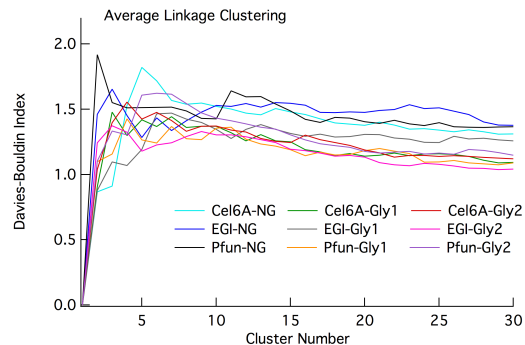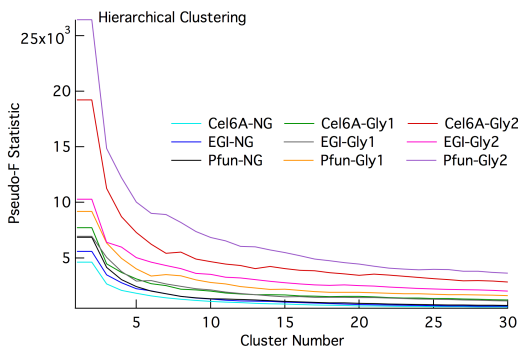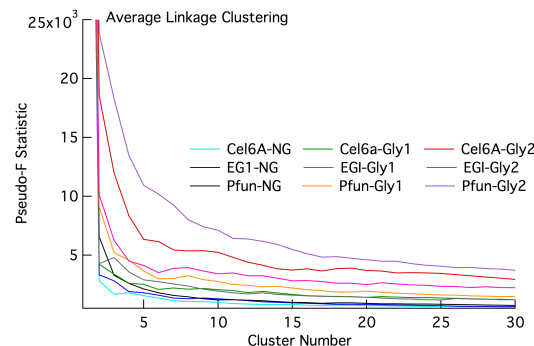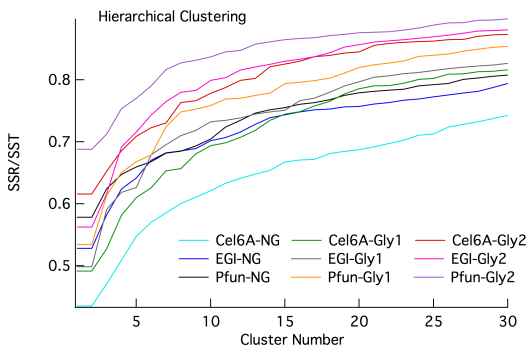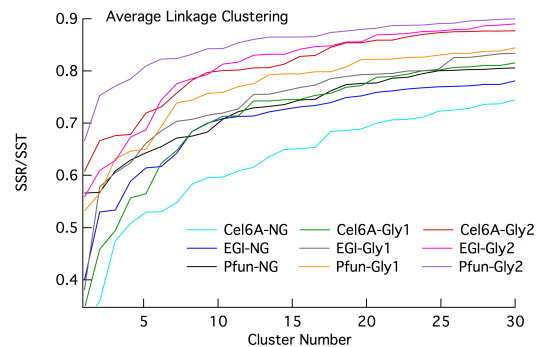

Supplement: Figure S8 — Sequence alignments for each domain from the four datasets: eukaryote GH7/CBM1, CBM1/GH6 and bacterial CBM1/GH6 and GH6/CBM1. (PDF) [file pone.0048615.s008.pdf]
