# Supplementary material for: Cellulase Linkers Are Optimized Based on Domain Type and Function: Insights from Sequence Analysis, Biophysical Measurements, and Molecular Simulation
Source: PLoS One. 2012 Nov 6;7(11):e48615. doi: 10.1371/journal.pone.0048615 (PMC3490864; doi:10.1371/journal.pone.0048615)
Supplement: Information S1 — Supplementary methods and tables. (PDF) [file pone.0048615.s010.pdf]

Supplementary Information

Supplemenary Methods

For the REMD simulations, sufficient sampling was confirmed through evaluation of the transit number and the potential energy autocorrelation time as described by Abraham and Gready [1]. The transit number, the number of replica-exchange attempts required for a 95% probability that the maximum temperature is visited prior to the minimum, is a practical measure of the minimum number of exchange attempts necessary for a given replica scheme. To obtain the transit number, the estimated average exchange probability for each replica-scheme evaluated in this manuscript was compared to a range of transit numbers, which are a function of replica-scheme size and average exchange probabilities, determined by Abraham and Gready [1]. The average exchange probability can be estimated as in Equation S1 below.

$$P(exchange) = \min\left(1, \exp\left[-\frac{\varepsilon^2 N_{df}}{1+\varepsilon}\right]\right) \tag{S1}$$

Where 1+ε represents the constant ratio between temperatures in an exponentially distributed replica scheme, and N_{df} is the number of degrees of freedom, which is estimated as number of atoms in the simulation. Abraham and Gready recommend that a simulation exceed the transit number by at least two orders of magnitude in order to achieve sufficient mixing efficiency [1]. Table SI summarizes the transit number and subsequent exchange attempt recommendations for each simulation set performed here. Comparing the recommended exchange attempts with the performed number of exchange attempts, as shown in Table SH, the simulations should achieve sufficient mixing efficiency.

Additionally to make conformational sampling most efficient, successive exchange attempts should be made so as to ensure measurements are independent. This can be achieved by choosing an exchange period approximately as large as the autocorrelation time of the potential energy, as discussed by Abraham and Gready [1]. Here, the autocorrelation of the potential energy was calculated as:

$$c(t) = \frac{1}{c_o(N-t)} \sum_{j=1}^{N-t} \left(U_j - \overline{U}\right)\left(U_{j+t-1} - \overline{U}\right) \tag{S2}$$

where c_o and $c(t)$ are the autocorrelation function at time 0 and t, respectively, N is the number of sample points, and U is the potential energy. The autocorrelation function, $c(t)$, was analyzed through block averaging over the 15 ns simulation trajectory. The approximate exhange interval was calculated by averaging the cumulative sums of $c(t)*\Delta t$ for each block. Autocorrelation data were truncated at 400 ps to avoid including noise dominance in the determination of the exchange interval. Ultimately, the average exchange interval should be roughly 3.5 ps based on simulation data. Simulations performed here use an interval of 3 ps ensuring sufficient mixing efficiency and data decorrelation.

Supplementary Tables

Table SA: GH Family 7 eukarya – (GH7/CBM1)

GenBank	Organism
AAA34212.1	*Hypocrea jecorina*
AAA65587.1	*Fusarium oxysporum*
AAD41096.1	*Volvariella volvacea V14*
AAF04492.1	*Aspergillus niger CBS 513.88*
AAF36391.1	*Hypocrea lixii FP 108*
AAK95563.1	*Lentinula edodes STAMETS CS-2*
AAM54070.1	*Emericella nidulans*
AAO42612.1	*Gibberella zeae K59*
AAQ21382.1	*Hypocrea rufa AS 3.3711*
AAQ38146.1	*Chrysosporium lucknowense*
AAQ76092.1	*Hypocrea rufa AS 3.3711*
AAS82856.1	*Nectria haematococca mpVI*
AAS82857.1	*Gibberella avenacea*
AAS82858.1	*Gibberella pulicaris 2*
AAT64006.1	*Volvariella volvacea*
AAT84320.1	*Chaetomium thermophilum var. thermophilum DSM1495*
AAT99321.1	*Penicillium occitanis Pol6*
AAV65115.1	*Penicillium chrysogenum FS010*
AAW64926.1	*Chaetomium thermophilum CT2*
AAW68437.2	*Aspergillus terreus SUK-1*
AAX28897.1	*Hypocrea jecorina PTCC 5142*
AAX60001.1	*Fusarium venenatum 5*
AAX60003.1	*Fusarium poae*
ABM90986.1	*Hypocrea pseudokoningii*
ABY56790.1	*Penicillium decumbens 114-2*
ACE60553.1	*Penicillium oxalicum F67*
ACF93800.1	*Hypocrea virens UKM1*
ACH68455.1	*Trichoderma sp. SSL*
ACH96125.1	*Trichoderma sp. XST1*
ACJ15337.1	*Penicillium decumbens L-06*
ACS32299.1	*Penicillium oxalicum*
ACV95805.1	*Penicillium decumbens 114-2*
ACX42576.1	*Hypocrea rufa*
ACZ06578.1	*Hypocrea rufa*
ACZ34302.1	*Trichoderma longibrachiatum FU05*
ADH04808.1	*Hypocrea lixii A25-2*
ADM08177.1	*Hypocrea jecorina M5*
ADN88316.1	*Neolentinus lepideus*
ADO21450.1	*Aspergillus niger nl-1*

ADR78837.1	*Aspergillus terreus MS-31*
ADX60067.1	*Penicillium funiculosum*
AEC03714.1	*Trichoderma longibrachiatum*
AEF33951.1	*Penicillium oxalicum M*
AEF58998.1	*Aspergillus niger AN524*
BAA09785.1	*Humicola grisea var. thermoidea IFO9854*
BAA25183.1	*Aspergillus aculeatus F-50*
BAA36215.1	*Hypocrea rufa MC300-1*
BAA76363.1	*Irpex lacteus MC-2*
BAA76364.1	*Irpex lacteus MC-2*
BAA76365.1	*Irpex lacteus MC-2*
BAC81967.1	*Athelia rolfsii*
BAD16575.1	*Irpex lacteus MC-2*
BAJ07534.1	*Flammulina velutipes Fv-1*
CAA02835.1	*Neurospora crassa*
CAA35159.1	*Humicola grisea var. thermoidea*
CAA37878.1	*Hypocrea rufa*
CAA38275.1	*Phanerochaete chrysosporium BKM-1767*
CAA41780.1	*Penicillium janthinellum Biourge IMET 43733 (C41)*
CAA43059.1	*Trichoderma longibrachiatum CECT 2606*
CAA49596.1	*Hypocrea koningii G-39*
CAA80253.1	*Phanerochaete chrysosporium*
CAA82761.1	*Phanerochaete chrysosporium*
CAA90422.1	*Agaricus bisporus D649*
CAC85737.1	*Penicillium funiculosum*
CAD79778.1	*Acremonium thermophilum*
CAD79779.1	*Chaetomium thermophilum*
CAD79782.1	*Thielavia australiensis*
CAD79790.1	*Exidia glandulosa*
CAD79795.1	*Chaetomidium pingtungium*
CAD79797.1	*Scytalidium thermophilum*
CAH10320.1	*Hypocrea jecorina L27*
CAK18798.1	*Pleurotus sp. 'Florida' FLORIDA N001*
CAK18799.1	*Pleurotus sp. 'Florida' FLORIDA N001*
CAK18800.1	*Pleurotus sp. 'Florida' FLORIDA N001*
CAM98445.1	*Acremonium thermophilum ALKO4245*
CAM98448.1	*Chaetomium thermophilum ALKO4265*
CAP61105.1	*Podospora anserina S mat+*
CAP85526.1	*Penicillium chrysogenum Wisconsin 54-1255*
EAA66593.1	*Aspergillus nidulans FGSC A4*

Table SB: GH Family 6 eukarya – (CBM1/GH6)

GenBank	Organism
ABY52798.1	*Piromyces rhizinflatus*
AAR08200.1	*Neocallimastix frontalis*
ADO33719.1	*Neocallimastix sp. AF-CTS-CDN1*
AAC09228.1	*Orpinomyces sp. PC-2*
AAQ09256.1	*Neocallimastix sp. W-1*
AAQ93324.1	*Neocallimastix frontalis*
ABY52793.1	*Neocallimastix patriciarum*
AAD41097.1	*Volvariella volvacea*
BAH08702.1	*Coprinopsis cinerea*
BAH59082.1	*Coniophora puteana*
AAB32942.1	*Phanerochaete chrysosporium*
BAG48183.1	*Irpex lacteus*
AAF35251.1	*Trametes versicolor*
BAF80327.1	*Polyporus arcularius*
AAL15037.1	*Lentinus sajor-caju*
AAK28357.1	*Lentinula edodes*
AAK95564.1	*Lentinula edodes*
AAA50607.1	*Agaricus bisporus*
AAT64008.1	*Volvariella volvacea*
ADN88317.1	*Neolentinus lepideus*
XP_360146.1	*Magnaporthe oryzae 70-15*
AAU05379.2	*Trichoderma parceramosum*
AAK01367.1	*Hypocrea koningii*
AAQ76094.1	*Trichoderma viride*
AAA34210.1	*Hypocrea jecorina*
ADC83999.1	*Hypocrea jecorina*
ADJ10628.1	*Trichoderma viride*
ABF56208.1	*Hypocrea koningii*
ABG48766.1	*Hypocrea koningii*
ACH96126.1	*Trichoderma sp. XST1*
ACZ34301.1	*Trichoderma longibrachiatum*
ADZ99361.1	*Phialophora sp. CGMCC 3328*
ADX86895.1	*Penicillium decumbens*
AAL78165.2	*Talaromyces emersonii*
ABF50873.1	*Emericella nidulans*
CAK41068.1	*Aspergillus niger*
AAE50824.1	Sequence 1 from patent US 6127160
ACH91035.1	*Penicillium funiculosum*
AAA65585.1	*Fusarium oxysporum*
AAQ72468.1	*Gibberella zeae*
CAH05678.1	*Stilbella annulata*

CAD70733.1	*Neurospora crassa*
BAB39154.1	*Humicola insolens*
AAW64927.1	*Chaetomium thermophilum*
CAH05669.1	*Chaetomium thermophilum*
ABT06036.1	Sequence 2 from patent US 7220565
CAP60942.1	*Podospora anserina S mat+*

Table SC: GH Family 6 bacteria with CBM2 N-terminal to GH – (CBM2/GH6)

GenBank	Organism
AAA23084.1	*Cellulomonas fimi*
AAA62211.1	*Thermobifida fusca*
AAE63612.1	*Sequence 2 from patent US 6207436*
ABP56033.1	*Salinispora tropica CNB-440*
ABV99773.1	*Salinispora arenicola CNS-205*
ACU36136.1	*Actinosynnema mirum DSM 43827*
ACU75529.1	*Catenulispora acidiphila DSM 44928*
ACV08202.1	*Jonesia denitrificans DSM 20603*
ACZ32147.1	*Xylanimonas cellulosilytica DSM 15894*
ADG75795.1	*Cellulomonas flavigena DSM 20109*
ADG89357.1	*Thermobispora bispora DSM 43833*
ADH67869.1	*Nocardiopsis dassonvillei subsp. Dassonvillei DSM 43111*
ADH68920.1	*Nocardiopsis dassonvillei subsp. Dassonvillei DSM 43111*
ADJ46954.1	*Amycolatopsis mediterranei U32*
ADL48574.1	*Micromonospora aurantiaca ATCC 27029*
ADW07422.1	*Streptomyces flavogriseus ATCC 33331*
AEB46944.1	*Verrucosispora maris AB-18-032*
AEE47298.1	*Cellulomonas fimi ATCC 484*
AEI13217.1	*Cellvibrio gilvus ATCC 13127*
AEN08184.1	*Streptomyces sp. SirexAA-E*
BAC69564.1	*Streptomyces avermitilis MA-4680*
BAJ21449.1	*Neisseria sicca*
CAA20645.1	*Streptomyces coelicolor A3(2)*
CBG68832.1	*Streptomyces scabiei 87.22*
CBG75944.1	*Streptomyces scabiei 87.22*

Table SD: GH Family 6 bacteria with CBM2 C-terminal to GH – (GH6/CBM2)

GenBank	Organism
CBA15018.1	*Xanthomonas albilineans*
CBJ53390.1	*Ralstonia solanacearum CFBP2957*
CAD17734.1	*Ralstonia solanacearum GMI1000*
AEG71050.1	*Ralstonia solanacearum Po82*
CAQ58880.1	*Ralstonia solanacearum IPO1609*
ACA11602.1	*Xylella fastidiosa M12*

AAF84076.1	*Xylella fastidiosa 9a5c*
AAO28402.1	*Xylella fastidiosa Temecula1*
ACB91997.1	*Xylella fastidiosa M23*
ADG74793.1	*Cellulomonas flavigena DSM 20109*
AAC06388.1	*Thermobifida fusca*
ADH69587.1	*Nocardiopsis dassonvillei subsp. Dassonvillei DSM 43111*
AEB43963.1	*Verrucosispora maris AB-18-032*
ADJ45769.1	*Amycolatopsis mediterranei U32*
ACU36111.1	*Actinosynnema mirum DSM 43827*
ACU37123.1	*Actinosynnema mirum DSM 43827*
AEC53449.1	*Actinosynnema pretiosum subsp auranticum*
ACZ89121.1	*Streptosporangium roseum DSM 43021*
ADG89529.1	*Thermobispora bispora DSM 43833*
ACU75402.1	*Catenulispora acidiphila DSM 44928*
AAC60491.1	*Micromonospora cellulolyticum*
ADL44187.1	*Micromonospora aurantiaca ATCC 27029*
ACV08399.1	*Jonesia denitrificans DSM 20603*

Table SE: GH Family 6 eukarya - ruminal fungi from CBM1/GH6

GenBank	Organism
AAC09228.1	*Orpinomyces sp. PC-2*
AAQ09256.1	*Neocallimastix sp. W-1*
AAQ93324.1	*Neocallimastix frontalis*
AAR08200.1	*Neocallimastix frontalis*
ABY52793.1	*Neocallimastix patriciarum*
ABY52798.1	*Piromyces rhizinflatus*
ADO33719.1	*Neocallimastix sp. AF-CTS-CDN1*

Table SF: GH Family 6 bacteria - proteobacteria from GH6/CBM2

GenBank	Organism
AAF84076.1	*Xylella fastidiosa 9a5c*
AAO28402.1	*Xylella fastidiosa Temecula1*
ACA11602.1	*Xylella fastidiosa M12*
ACB91997.1	*Xylella fastidiosa M23*
AEG71050.1	*Ralstonia solanacearum Po82*
CAD17734.1	*Ralstonia solanacearum GMI1000*
CAQ58880.1	*Ralstonia solanacearum IPO1609*
CBA15018.1	*Xanthomonas albilineans GPE PC73*
CBJ53390.1	*Ralstonia solanacearum CFBP2957*

Table SG. Student's t-test p-values for the comparison of linker lengths from each of the four datasets.

	**t-test p-values**
**GH7-CBM1 vs CBM1-GH6**	9.71E-11
**GH7-CBM1 vs CBM2-GH6**	0.07
**GH7-CBM1 vs GH6-CBM2**	0.31
**CBM1-GH6 vs CBM2-GH6**	0.03
**CBM1-GH6 vs GH6-CBM2**	0.13
**CBM2-GH6 vs GH6-CBM2**	0.94

Table SH. R^2 values for the trend lines for combined serine and threonine content as a function of linker length and proline content as a function of linker length shown in Figure 5.

	Serine+Threonine R^2	Proline R^2
GH7/CBM1	0.65	0.15
CBM1/GH6	0.72	0.02
CBM2/GH6	0.47	0.73
GH6/CBM2	0.56	0.87

Table SI: Comparison of genera found in both eukaryotic sequence sets, and both bacterial sequence sets. Considerable overlap is present, suggesting gene or organisms sampling bias does not account for the observed differences seen linker length.

**Eukaryote**	
66%	of CBM1/GH6 genera found in GH7/CBM1
87%	of GH7/CBM1 genera found in CBM1/GH6

**Bacteria**	
52%	of CBM2/GH6 genera found in GH6/CBM2
93%	of GH6/CBM2 genera found in CBM2/GH6

Table SJ. System parameters for REMD simulations.

Cellulase Linker with Extent of Glycosylation	System Size [atoms]	Attempted Swaps	Temperature Range [K]	Number of Replicas
P. funiculosum Cel7A, no glycan	410	50,000	300-550	12
P. funiculosum Cel7A, 1 glycan	935	80,000	300-600	20
P. funiculosum Cel7A, 2 glycans	1460	120,000	300-650	28
T. reesei Cel6A, no glycan	540	60,000	300-550	16
T. reesei Cel6A, 1 glycan	1024	80,000	300-600	20
T. reesei Cel6A, 2 glycans	1486	120,000	300-650	28
T. reesei Cel7B, no glycan	341	50,000	300-550	12
T. reesei Cel7B, 1 glycan	671	70,000	300-600	16
T. reesei Cel7B, 2 glycans	986	80,000	300-600	20

Table SK. Transit number and recommended exchange attempts for each replica-exchange scheme.

Cellulase Linker with Extent of Glycosylation	Temperature Ratio (1+e)	Exchange Probability	Transit Number	Recommended Exchange Attempts
P. funiculosum Cel7A, no glycan	1.057	0.288	200	20000
P. funiculosum Cel7A, 1 glycan	1.037	0.288	500	50000
P. funiculosum Cel7A, 2 glycans	1.029	0.302	600	60000
T. reesei Cel6A, no glycan	1.041	0.414	200	20000
T. reesei Cel6A, 1 glycan	1.037	0.256	400	40000
T. reesei Cel6A, 2 glycans	1.029	0.296	700	70000
T. reesei Cel7B, no glycan	1.057	0.355	100	10000
T. reesei Cel7B, 1 glycan	1.047	0.239	300	30000
T. reesei Cel7B, 2 glycans	1.037	0.269	400	40000

Supplementary Reference

1. Abraham MJ, Gready JE (2008) Ensuring mixing efficiency of replica-exchange molecular dynamics simulations. Journal of Chemical Theory and Computation 4: 1119-1128.
